# The inhibitory effect of curcumin via fascin suppression through JAK/STAT3 pathway on metastasis and recurrence of ovary cancer cells

**DOI:** 10.1186/s12905-020-01122-2

**Published:** 2020-11-19

**Authors:** Mi Ju Kim, Ki-Su Park, Kyoung-Tae Kim, Eun Young Gil

**Affiliations:** 1 Department of Obstetrics and Gynecology, Kyungpook National University Hospital, Kyungpook National University School of Medicine, 130 Dongdeok-ro, Jung-gu, Daegu, 41944 Republic of Korea; 2 Department of Neurosurgery, School of Medicine, Kyungpook National University, Daegu, Republic of Korea

**Keywords:** Ovary cancer, Curcumin, Fascin, JAK/STAT3 pathway

## Abstract

**Background:**

Fascin is an actin-binding protein and highly expressed in ovarian cancer cells. It is associated with metastasis of cancer and may be a useful prognostic factor. Anticancer activity of curcumin is related to its effect on several signaling mechanisms. Although there have been many reports regarding the anticancer properties of curcumin, its inhibitory effects on migration and invasion of ovarian cancer cells, particularly in the context of fascin expression, have not been reported. The purpose of this study was to investigate the effect of curcumin on fascin expression in ovarian cancer cells and to propose a possible mechanism for the anticancer activity of curcumin through reduced fascin expression.

**Methods:**

SKOV3, human epithelial ovary cancer cell line, was cultured with curcumin at various dose and duration. The fascin was quantified using cell viability test and Western blot. To determine the effect of curcumin on the upstream pathway of fascin expression, the signal transducer and activator of transcription 3 (STAT3) was analyzed by sandwich-ELISA. Attachment assay, migration assay and invasion assay were analyzed to approve the change of cellular invasiveness of ovary cancer after curcumin. To determine the morphological changes of ovarian cancer cells by curcumin, immunofluorescence was performed.

**Results:**

MTS assays showed that cell viability was different at various concentration of curcumin, and as concentration increased, cell viability tended to decrease. Curcumin appears to suppress fascin expression, even with a minimal concentration and short exposure time. Also, curcumin may suppress fascin expression in ovarian cancer cells through STAT3 downregulation. The attachment assay, migration assay and invasion assay of the ovarian cancer cells exhibited a statistically significant decrease. Immunofluorescence revealed a change of cell shape from a typical form of uninfluenced cells to a more polygonal appearance, with a significant reduction in filopodia formation.

**Conclusions:**

Curcumin reduces fascin expression through JAK/STAT3 pathway inhibition, which interferes with the cellular interactions essential for the metastasis and recurrence of ovarian cancer cells. Higher curcumin concentrations and longer exposure times concomitantly decreased fascin expression.

## Background

Ovarian cancer is one of the most common neoplasms associated with a high mortality rate among women worldwide [1]. Epithelial ovarian cancer occurs from the malignant transformation of the ovarian surface epithelium and spreads throughout the peritoneal mesothelium [2]. The symptoms of ovarian cancer are nonspecific and vague in many cases, resulting in diagnosis at an advanced stage. The treatment for advanced stage disease includes surgery followed by platinum-based chemotherapy. Several molecular therapies have been introduced that target pathways mediated by p53, lysophosphatidic acid, the BCL-2 family, epidermal growth factor receptor, and the vascular endothelial growth factor receptor [3–9]. Disease recurrence and mortality rate are associated with the degree of metastasis. The metastasis of tumor cells involves cell adhesion, migration, and invasion, and requires cellular skeletal disruption [10].

Fascin is an actin-binding protein that structurally reinforces the cytoplasmic microfilament bundle, which is a component of cell architecture, movement, and drives cell adhesion, migration, and invasion [11]. Several studies have reported high fascin expression associated with increased mortality in breast, colon, and pancreatic cancers, and with metastasis in colorectal and gastric cancers [12, 13]. Recent studies have suggested that fascin is highly expressed in ovarian cancer cells. It is also associated with metastasis and may be a useful prognostic factor [14]. Especially in non-epithelial ovary cancer, fascin being involved in the early phase of metastasis in vivo [11].

Curcumin (diferuloylmethane) is the principal curcuminoid of turmeric, an Indian spice with a yellowish color, derived from the *Curcuma longa Linn* plant [15]. Curcumin has anti-inflammatory, antioxidant, and anti-infective properties and its use is currently being investigated. Numerous research groups have been analyzing the anticancer properties of curcumin, in particular, its ability to inhibit cancer cell adhesion and migration [16–19]. Interestingly, curcumin has been shown to exhibit activity in stem cells of colorectal, pancreatic, breast, brain, and head and neck cancers [20–22]. Its anticancer activity is related to its effect on several signaling mechanisms, including the inhibition of transcription factors, proteases, protein kinases, inflammatory cytokines, and their respective signaling pathways [23].

Although there have been many reports regarding the anticancer properties of curcumin, its inhibitory effects on migration and invasion of ovarian cancer cells, particularly in the context of fascin expression, have not been reported. Therefore, the purpose of this study was to investigate the effect of curcumin on fascin expression in ovarian cancer cells and to propose a possible mechanism for the anticancer activity of curcumin through reduced fascin expression.

## Methods

### Cell culture

Cell lines were derived from the SKOV3 human epithelial ovary cancer cell line (ATCC, Manassas, VA, United States) [24]. Cells were cultivated in Dulbecco’s Modified Eagle Medium containing 10% heat-inactivated fetal bovine serum, 100 U/ml penicillin, and 100 µg/ml streptomycin. Cells were cultured at 37 °C in a humidified incubator with 5% CO_2_.

### Cell viability assay

Cell viability of SKOV3 cells after curcumin was determined MTS assay kit (CellTiter 96 Aqueous One Solution, Promega) [25]. SKOV3 cells were seeded and allowed to adhere overnight. Then, cells were incubated in FBS-free media containing desired concentrations of curcumin (0, 10, 20, 30, 40, and 50 μM) dissolved in dimethyl sulfoxide. MTS reagent was then added to each of the wells for various times (6, 24, 48, and 72 h). The optical density was measured at 490 nm with a microplate spectrophotometer.

### Western blot analysis

Western blot analysis was performed to quantify a fascin expression. SKOV3 cells were incubated in FBS-free media containing 0 and 10 μM curcumin for 6 h [25]. Then, cells were collected after 6, 12, and 24 h. The cell lysate was centrifuged at 13,000 rpm for 30 min at 4 °C, and the proteins were quantified by the bicinchoninic acid protein assay (Sigma, St. Louis, MO, USA). Protein samples were separated on 10% SDS–polyacrylamide electrophoresis gels and then transferred to nylon membranes (Millipore, Bedford, MA, USA). The membranes were blocked with 5% non-fat dry milk in Tris-buffered saline containing 0.1% Tween-20 for 1 h at room temperature. After incubation with a 1:1000 dilution of mouse monoclonal anti-Fascin (Santa Cruz, CA, USA) at 4 °C overnight, peroxidase-conjugated secondary antibody was added for 1 h at room temperature. The fluorescent signal was detected using an enhanced chemiluminescence system (Amersham Biosciences, Buckinghamshire, England). The immunoreactive band densities were calculated by image J software, version 1.46r, computer-assisted image analyzer (National institutes of Health, USA).

### Sandwich ELISA

To determine the effect of curcumin on fascin expression, the signal transducer and activator of transcription 3 (STAT3) was analyzed by sandwich-ELISA [25]. STAT3 is known as a transcription factor related to a master regulator of ovary cancer cells that is essential for maintaining a tumor’s initiating capacity and ability to invade the normal tissue. After SKOV3 cells were incubated in FBS-free media containing 10 μM curcumin. Cells were collected after 6, 12, and 24 h and lysed with an ice-cold lysis buffer solution. The concentrations and phosphorylation status of the transcription factor STAT3 were determined using a sandwich-ELISA kit (PathScan® Phospho-Stat3 (Tyr705) Sandwich-ELISA Antibody Pair #7146; Cell Signaling Technology Inc., Danvers, MA, USA). After coating the microplate wells, 100μL of the respective lysates were added to each well and incubated at 37 °C for 2 h before the wells were washed, and a detection antibody was added for 1 h. Then, 100 μL of a secondary polyclonal antibody, conjugated to horseradish peroxidase, was added to each well. The plate was then incubated for 30 min at room temperature. Finally, a 200 μL of substrate solution (Tetramethylbenzidine) was incubated in each well for 10 min at 37 °C. The reaction was terminated by adding 100 μL of stop solution (2 N sulfuric acid). The color in the wells changed from blue to yellow. The optical density of each well was measured using a microplate reader (VERSA max, Molecular Device Inc., CA, USA) set to 450 nm.

### Attachment assay

Ninety-six well plates were incubated at 4 °C overnight with laminin at 10 μg/ml [25]. The unbound sites were blocked with 0.1% BSA for 1 h. SKOV3 cells, which were temporarily treated with 10 and 20 μM curcumin for 6 h, were seeded at a density of 5 × 10^4^ cells/well on laminin or BSA-coated plates. The cells were allowed to adhere for 6, 12, and 24 h at 37 °C in a humidified incubator supplied with 5% CO_2_. After washing with PBS, the remaining cells were fixed with 4% paraformaldehyde for 10 min and then stained with 5% crystal violet for 20 min at room temperature. Cells were solubilized with 1% SDS and the absorbance of each well was measured using a microplate reader (VERSA max, Molecular Device Inc., CA, USA) set to 595 nm.

### Cell migration assay

SKOV3 cells were plated in 6-well dishes at a density of 0.3 × 10^6^ cells/well and were allowed to attach and reach 80% subconfluency [25]. Thereafter, they were incubated with a starvation medium containing 10 and 20 μM curcumin for 6 h. A scratch was formed through the cell monolayer. Cells were photographed using an Axioplan-2 epifluorescence microscope (Carl Zeiss Vision GmbH, Munchen, Germany) after 6, 12, and 24 h. Areas of the scratch were counted with image J, version 1.46r, computer-assisted image analyzer (National institutes of Health, USA).

### Cell invasion assay

Cell invasion was evaluated by a Matrigel-coated modified Boyden chamber (Biocoat™ Matrigel™ Invasion Chamber; Becton Dickinson GmbH, Heidelberg, Germany) [25]. Approximately 0.25 × 10^5^ control and SKOV3 cells treated with 10 and 20 μM curcumin for 6 h were seeded into the upper well of the chamber containing 500 μL serum free culture medium. A 500 μL culture medium with 10% FBS was added to the bottom of the well. After 6, 12, and 24 h, non-invading cells in the top chamber were removed gently with a cotton swab, cells on the bottom of the chamber were fixed with 4% paraformaldehyde for 15 min at room temperature, and then stained with 0.5% crystal violet at room temperature for 10 min. After the crystal violet was rinsed with distilled water, cells were photographed using an Axioplan-2 epifluorescence microscope (Carl Zeiss Vision GmbH, Munchen, Germany).

### Immunofluorescence

To determine the morphological changes of ovarian cancer cells by curcumin, immunofluorescence was performed [25]. SKOV3 cells were seeded at a density of 5000 cells/well in a 96-well plate and allowed to adhere overnight. After temporary exposure to 0 (control) and 10 μM curcumin for 6 h, the cells were washed three times with PBS. After 6 and 24 h, cells were fixed in 4% paraformaldehyde (PFA) for 10 min and permeabilized for 5 min with 0.5% Triton X-100 in PBS at room temperature. Subsequently, cells were incubated with an anti-Fascin 1 monoclonal antibody (1:100, sc-46675, Santa Cruz Biotechnology, Santa Cruz, CA, USA) for 1 h. After washing, cells were then incubated with Alexa Fluor 594-conjugated goat anti–mouse IgG secondary antibodies (Molecular Probes, Eugene, OR, USA) for 30 min at room temperature. The images were viewed on a computer monitor using a Zeiss Plan-Apochromat 40 × objective (Carl Zeiss Vision GmbH, Munchen, Germany).

### Statistical analysis

All statistical comparisons were calculated using SPSS 22.0 software (SPSS, Inc., an IBM Company, Chicago, IL, USA). Data were expressed as mean ± standard error of the mean. Repeated measure ANOVA was used to compare groups. Null hypotheses of no difference were rejected if *p* values were less than 0.05.

## Results

### Cell viability tests with respect to curcumin concentration and exposure time

**Fig. 1:**
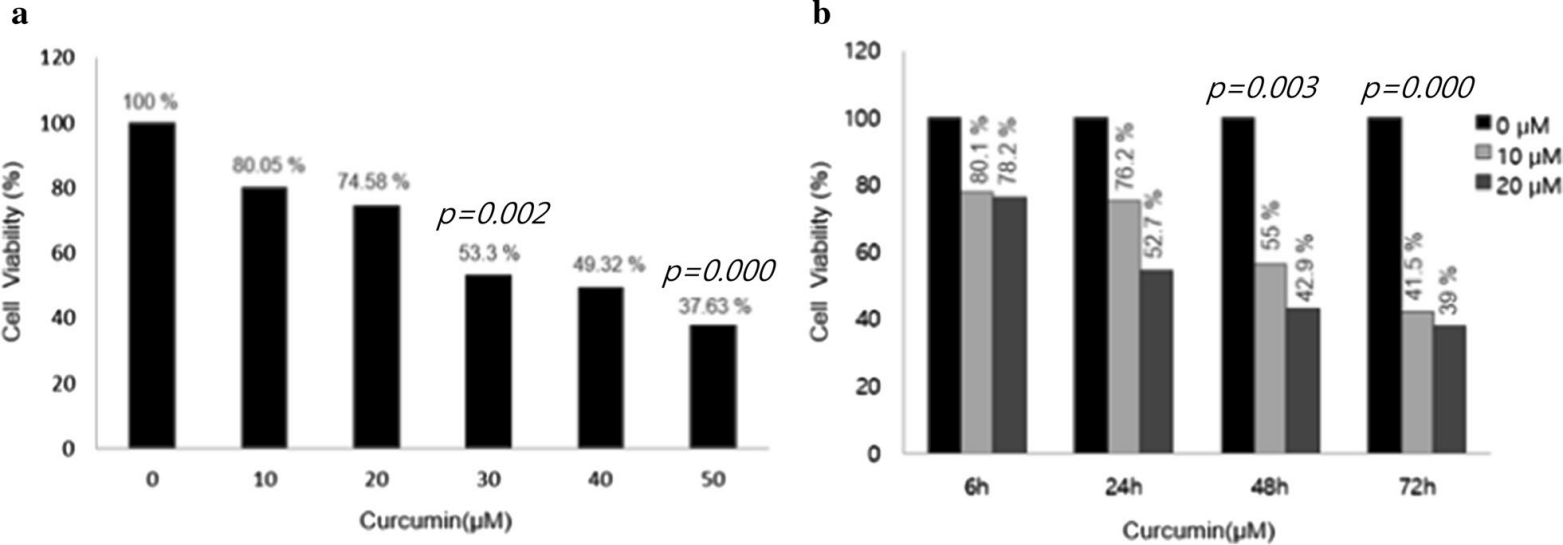
MTS assays showing that cell viability was different at various concentration of curcumin, and as concentration increased, cell viability tended to decrease. **a** At 10 μM/L concentrations of curcumin, cell viabilities showed minimal decrease (80.05%). And at 30 μM/L concentrations of curcumin, cell viabilities decreased to 53.2% (*p* = 0.002), at 50 μM/L concentrations of curcumin, cell viabilities decreased to 37.63% (*p* = 0.000). **b** The cell viabilities at 10 and 20 μM/L concentrations of curcumin for 48 h were 55% and 42.9% (*p* = 0.003). For 72 h, the cell viabilities at 10 and 20 μM/L concentrations of curcumin were 41.5% and 39% (*p* = 0.000)

SKOV3 cells were treated with different concentrations of curcumin (0, 10, 20, 30, 40, and 50 μM) for 12 h. MTS assays showed that cell viability was different at various concentration of curcumin, and as concentration increased, cell viability tended to decrease. However, cell viability after 10 and 20 μM curcumin did not show significant decreases compared with the control group (10 μM: 80.05% and 20 μM/L: 74.58%). When the concentration was increased to 30 μM, the cell viability was reduced by more than 50 percent compared with controls (Fig. 1a). SKOV3 cells were treated with 10 and 20 μM curcumin for different times (6, 24, 48 and 72 h). At all concentrations, the cell viability decreased by prolonged exposure time and the rate of decrease differed with concentration and time. The cell viabilities at 10 and 20 μM curcumin for 6 h revealed minimal decreases compared with the control group (10 μM: 80.1% and 20 μM: 78.2%). After 24 h, cell viability revealed consistent decreases below 75% (Fig. 1b). Consequently, according to the cell viability assays, 10 and 20 μM curcumin and 6 h were chosen as the minimally lethal concentrations and exposure time.

### Curcumin suppresses fascin expression by STAT3 inhibition in ovarian cancer cells

**Fig. 2:**
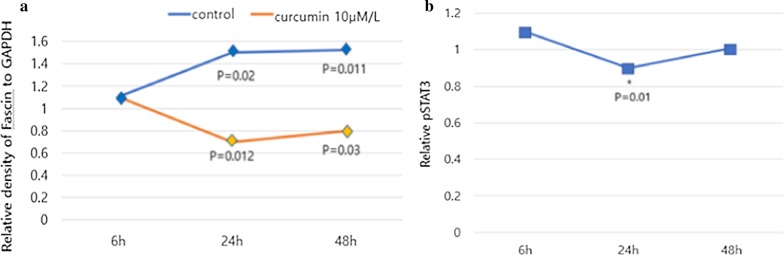
Western blot and sandwich ELISA. Suppression of fascin by curcumin inhibits migration and invasion of cancer cells via STAT3 downregulation. **a** Fascin expression decreased at 24 h (*p* = 0.012), but gradually increased after 24 h. **b** The change of STAT3 level decreased patterns of fascin expression at 24 h (*p* = 0.01)

When ovarian cancer cells were treated with 10 μM curcumin for 6 h, fascin expression decreased after 24 h (31.1%, *p* = 0.012) following treatment. Subsequently, it gradually recovered (Fig. 2a). Under the same conditions, STAT3 levels also reflected a temporarily decreased pattern of fascin expression at 24 h (29.7%, *p* = 0.010) (Fig. 2b). Therefore, curcumin appears to suppress fascin expression, even with a minimal concentration and short exposure time. Also, curcumin may suppress fascin expression in ovarian cancer cells through STAT3 downregulation.

### Suppression of fascin by curcumin inhibits the migration and invasion of cancer cells

**Fig. 3:**
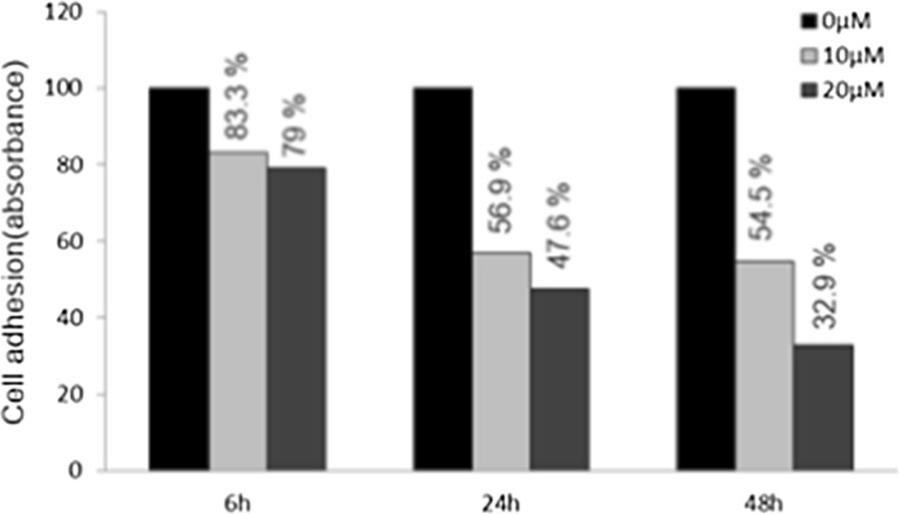
Attachment assay of SKOV3 cells treated with 10 and 20 μM curcumin for 6 h. Once the curcumin was washed out, the attachment assays were performed after 6, 24, and 48 h

**Fig. 4:**
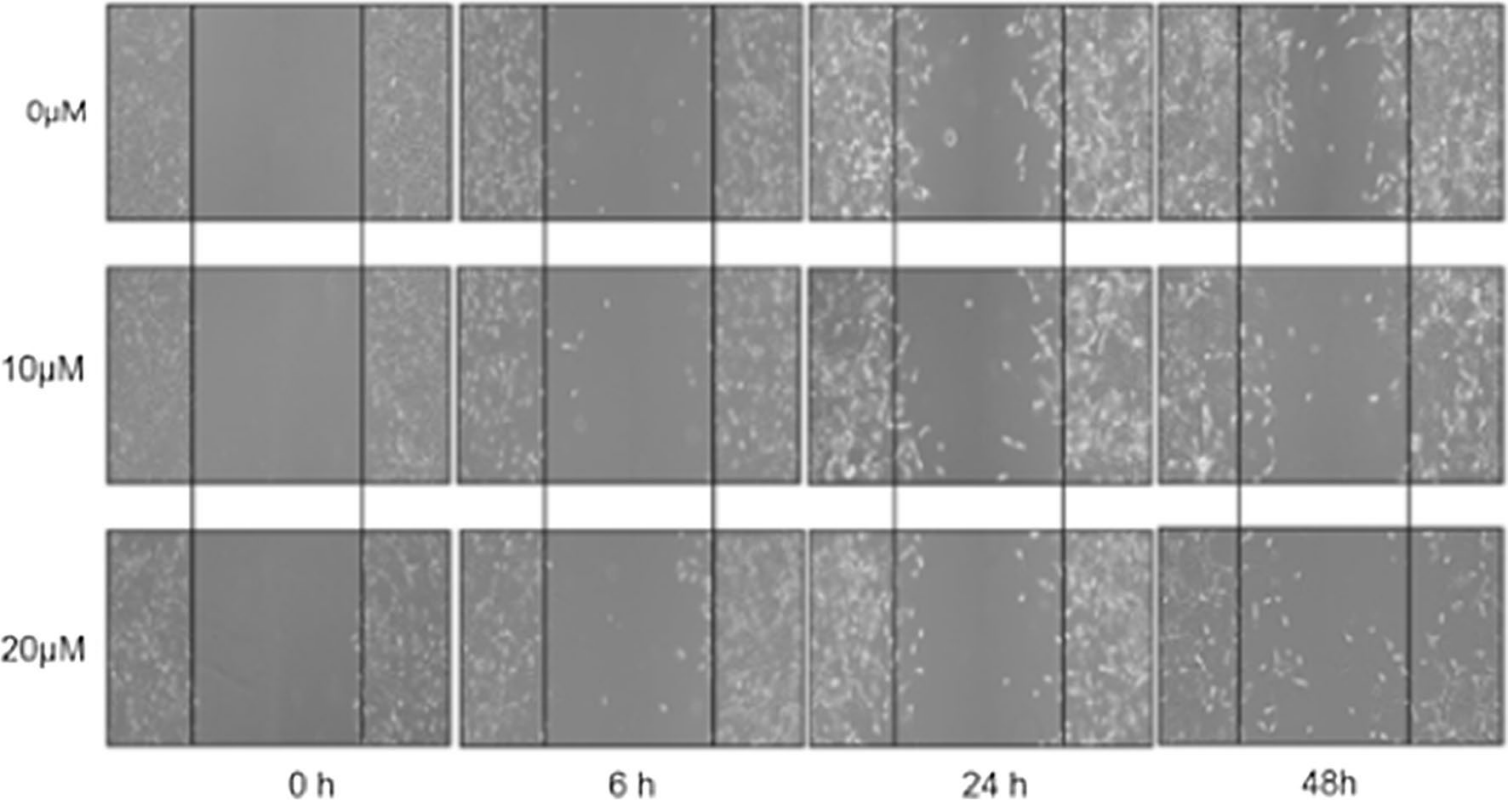
Cell migration assay. The migration ability of ovarian cancer cells revealed a significant decline after 6 h following curcumin pretreatment

**Fig. 5:**
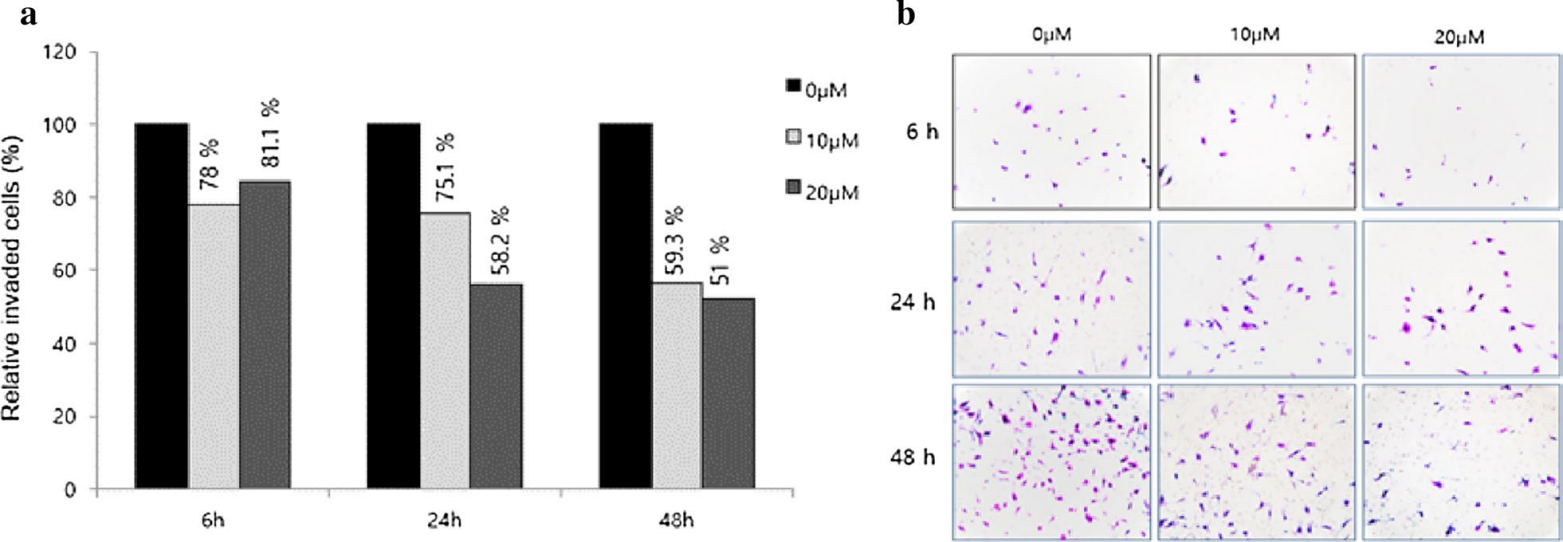
Transwell invasion assay. The invasion ability of ovary cancer cells resulted in a statistically significant decline after 24 h

SKOV3 cells were treated with 10 and 20 μM curcumin for 6 h, and then the curcumin was washed out. Attachment, migration and invasion assays were performed after 6, 24 and 48 h. The attachment assay of the ovarian cancer cells exhibited a statistically significant decrease after 6 h (10 and 20 μM curcumin at 6 h: 16.7%, 21%, *p* = 0.02. 0.01; 10 and 20 μM/L curcumin at 24 h: 43.1% and 52.4%, *p* < 0.001 and *p* < 0.001) (Fig. 3). The migration ability of ovarian cancer cells revealed a significant decline after 6 h and the percent closure was statistically different when compared to controls (10 μM and 20 μM at 12 h: 24.2% and 58.1%, *p* = 0.031 and *p* < 0.001; at 10 μM and 20 μM at 24 h: 25.0% and 54.3%, *p* = 0.002 and *p* < 0.001) (Fig. 4). The invasion ability of ovarian cancer cells using a transwell invasion assay revealed a statistically significant decline after 24 h (10 μM and 20 μM at 24 h: 24.9% and 41.8%, *p* = 0.001 and *p* < 0.001; 10 μM and 20 μM at 48 h: 40.7% and 49%, *p* = 0.001 and *p* < 0.001) (Fig. 5a, b).

### Curcumin changes cell shape in ovary cancer cells by suppression of fascin

**Fig. 6:**
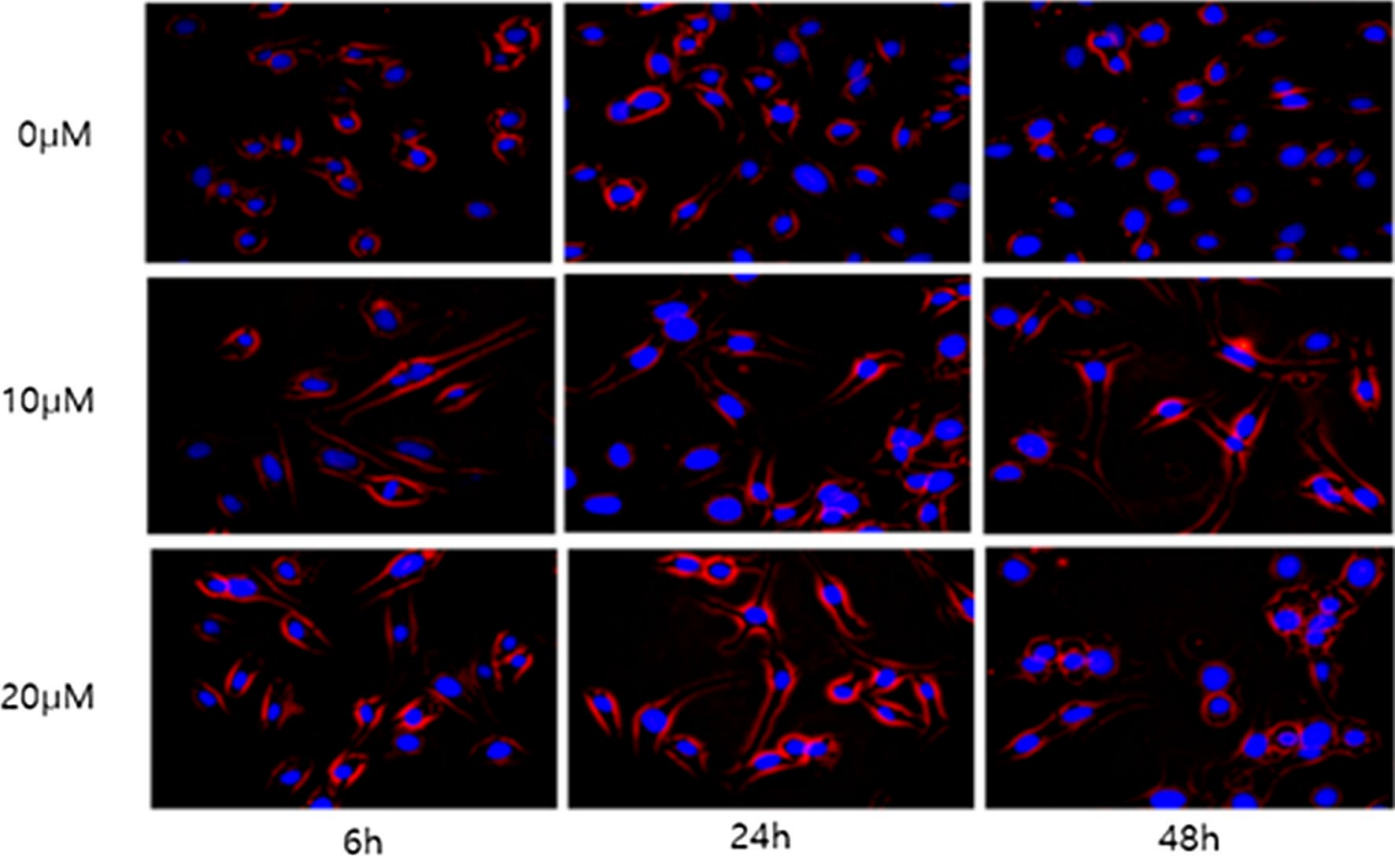
Immunofluorescence. Morphological changes of ovarian cancer cells were observed by immunofluorescence at 6, 24, and 48 h. Curcumin changes cell shape in ovary cancer cells by suppression of fascin

SKOV3 cells were treated with 10 μM curcumin for 6 h and the curcumin was then washed out. Morphological changes in the ovarian cancer cells were observed by immunofluorescence at 6, 24 and 48 h (Fig. 6). Immunofluorescence revealed a change of cell shape from a typical form of uninfluenced cells to a more polygonal appearance, with a significant reduction in filopodia formation. Furthermore, there was no recovery in the morphological change of the cells over time.

## Discussion

Our goal was to determine the effects of curcumin in ovarian cancer and the fascin pathway, affecting cellular interactions that are essential for recurrence and metastasis of cancer cells.

Cancer is known as a serious disease that impacts human life, and countless scholars have been trying to develop a cure for it. According to 2018 statistics, despite being outside the top 10 of newly diagnosed cancers, ovarian cancer is the fifth lethal cancer among women in the USA [1]. Ovarian cancer is usually at an advanced stage at the time of diagnosis, and it is also recognized as a cancer that is difficult to treat due to the development of drug resistance. Thus far, numerous studies have been published on ovarian cancer, many related to traditional surgery and chemotherapy. More recently, the scope of interest has been expanding into targeted therapy and hormone therapy. The presence of metastasis is a major obstacle that significantly impacts treatment and recurrence.

With respect to metastasis, the processes of cell adhesion, attachment, invasion and modification of filopodia are essential. Cytoskeletal and intercellular interactions between cancer cells and non-cancer, host stromal cells are also required [11]. In this step, multiple actin-binding proteins have a major role in the rearrangement of the actin-cytoskeleton complex of cells, and are essential to cancer cell invasion and cell rigidity modification [26]. The fascin pathway is important to cell structure and function [26], and represents a significant signaling pathway that is associated with cellular interactions during cancer progression [27, 28]. Fascin is an actin-binding protein and known to exist in the nervous system, vascular endothelial cells, genitourinary system, and gastrointestinal system. Fascin functions by producing filopodia and lamellopodia [27], and it is essential to cellular invasion, as it affects extracellular matrix cytoplasmic microfilaments. Several studies have reported a relationship between cancer occurrence and fascin expression. High fascin expression has been associated with increased mortality in breast, colorectal, and pancreatic cancers, and with metastasis in colorectal and gastric cancers [12, 13]. Recent studies have suggested that fascin is highly expressed in ovarian cancer cells, it is associated with metastasis, and may be a prognostic factor [14]. High fascin expression in cancer is associated with worse prognosis and a shorter disease-free interval [12]. The suppression of fascin may represent a key protein in cancer treatment and recurrence.

Curcumin (diferuloylmethane) is known as an Indian spice with a yellowish color that is derived from the *Curcuma longa Lin* plant [15]. People have been consuming curry for a long time, and it is widely enjoyed as a healthy food. Actually, curcumin exhibits anti-inflammatory, antioxidant, and anti-infective properties and its use is being investigated. Recent numerous studies revealed the anticancer properties of curcumin involve the inhibition of cancer cell adhesion and migration, which are essential for cancer cell survival [16–19]. Its anticancer effect is related to the attenuation of several signaling mechanisms, including the inhibition of transcription factors, proteases, protein kinases, inflammatory cytokines, and their respective signaling pathways [23]. Many investigators have demonstrated protective effects of curcumin on cancer stem cells from colorectal, pancreatic, breast, brain, and head and neck cancers [20–22]. Recently, many studies are also underway on the clinical application of curcumin's ani-cancer effect, and experimental results have been published in colon cancer, pancreatic cooler, etc., where merging therapy with curcumin alone or with other drugs may be effective.

Several previous reports have indicated that curcumin has efficacy in the treatment of ovarian cancer. Koroth et al. demonstrated that the curcumin derivatives, ST03 and ST08, induced ovarian cancer cell death by activating the intrinsic apoptotic pathway [29]. Liu et al. suggested that curcumin reduces cancer cell viability and enhances protective autophagy of ovarian cancer cells by inhibiting the AKT/mTOR/p70S6K signaling pathway [30]. McGuire et al. reported that fascin inhibition blocked ovary cancer metastasis in ovarian cancer cells and stromal cells [11]. At the present time, there are no studies regarding the effects of curcumin and fascin in ovarian cancer.

In the SKOV3 ovarian cancer cell line, which was treated with curcumin in an MTS assay, exposure to higher concentrations and greater exposure time resulted in decreased viability. Curcumin had a toxic effect on the ovarian cancer cells and it was clear that it affected cell survival. In this study, we decided to conduct experiments with less toxic concentrations, so that we set the minimum exposure concentration at 10 μM and exposure time at 6 h. Through cell attachment assays, the longer the curcumin exposure time, the higher the exposure concentration, and a statistically significant decrease in cell adhesion was observed. Based on the decrease in fascin density when the curcumin exposure time was longer, western blot analysis revealed that curcumin is involved in fascin inhibition and pSTAT3 levels analyzed through ELISA appeared similar to that of fascin. Therefore, curcumin has an inhibitory effect on STAT3 through the JAK/STAT3 signaling pathway, and STAT3 inhibition is also involved with the inhibition of fascin. The cell migration assay also indicated that cell closures were reduced when exposed to high concentrations of curcumin. Curcumin exposure also affected the formation of architecture and filopodia in the ovarian cancer cells. A higher curcumin exposure concentration and exposure time resulted in a greater effect.

The limit of this study is that it is an experiment using cell lines on the market, and it is thought that it will be necessary to compare the results of curcumin according to the type of cancer cells by cultivating ovarian cancer cells obtained from cancer patients in the future. It is also thought that it is necessary to conduct in vivo research through animal experiments using rat and to confirm that it can be applied to clinical trials.

## Conclusions

In this study, curcumin reduces fascin expression through JAK/STAT3 pathway inhibition, which interferes with the cellular interactions essential for the metastasis and recurrence of ovarian cancer cells. Higher curcumin concentrations and longer exposure times concomitantly decreased fascin expression.

## Data Availability

The datasets used and/or analyzed during the current study are available from the corresponding author on reasonable request.

## Abbreviations

JAK: Janus family tyrosine kinase
STAT3: Signal transducer and activator of transcription 3
ELISA: Enzyme-linked immunosorbent assay
FBS: Fetal bovine serum
